# Disposition and metabolism of 2′,2′”-Dithiobisbenzanilide in rodents following intravenous and oral administration and dermal application

**DOI:** 10.1016/j.toxrep.2020.07.006

**Published:** 2020-07-23

**Authors:** C. Edwin Garner, Christopher J. Wegerski, Melanie Doyle-Eisele, Jacob D. McDonald, J. Michael Sanders, Benjamin C. Moeller, Suramya Waidyanatha

**Affiliations:** a Lovelace Respiratory Research Institute, Albuquerque, New Mexico, USA; b Division of National Toxicology Program, National Institute of Environmental Health Sciences, Research Triangle Park, North Carolina, USA

**Keywords:** Metabolism, Disposition, Distribution, Absorption

## Abstract

2′,2′′′-Dithiobisbenzanilide (DTBBA) is a high-production-volume chemical used as a peptizing agent for rubber. The disposition and metabolism of [^14^C]DTBBA were determined in male and female rats and mice following oral (4, 40, or 400 mg/kg) and intravenous (IV) (4 mg/kg) administration and dermal application (0.4 or 4 mg/kg). [^14^C]DTBBA was well absorbed following oral administration (> 60%) and dermal application (∼40–50%) in rats and mice. Following oral administration, the majority of radioactivity was excreted in urine (29 − 70%) and feces (16 − 45%). Unlike rats, mice excreted ∼1-5% of the dose as exhaled CO_2_. The residual radioactivity in tissues was <1% in both species and sexes. The pattern of disposition following IV administration in male rats was similar to that following oral. When [^14^C]DTBBA was administered via IV to rats, a significant portion of the dose was recovered in bile (∼13%) suggesting that at least a portion of the dose recovered in feces following oral administration was likely the absorbed dose. The profiles of urine from rats and mice were similar and consisted of four major metabolites and three minor metabolites. The predominant metabolite in urine was the *S*-glucuronide of the thiol/sulfide cleavage product *N*-(2-mercaptophenyl)benzamide, which accounted for more than 50% of radioactivity in the radiochromatogram.

## Highlights

• 2′,2′′′-Dithiobisbenzanilide (DTBBA) is a chemical used as a peptizing agent for rubber.
• Humane exposure to DTBBA is possible via oral and dermal routes.
• DTBBA is well-absorbed in rodents following oral and dermal administration.
• Absorbed DTBBA was extensively metabolized and excreted mainly via urine.
• N-(2-mercaptophenyl)benzamide accounted for more than 50% of radioactivity in urine.

## 1 Introduction

2′,2′′′-Dithiobisbenzanilide (DTBBA) is a high-production-volume chemical used primarily as a peptizing agent for rubber. It reduces the viscosity of rubber and as a result facilitates the molding of rubber into various end products. The compound is mostly used in tires, tubes, remolds, and retreads, but is also present in other products such as belts, cables, hoses, rubber gloves, latex gloves, printing screen rollers, food processing equipment, aircraft de-icing equipment, automobile parts, toys, medical equipment and athletic shoes [1]. Occupational exposure to DTBBA in the rubber industry may occur through inhalation and dermal contact. Consumers may be exposed via orally to small amounts of DTBBA leached from rubber products used to transport or package food and from rubber and plastic products designed for sale to the consumer market. Currently, there is no data regarding the levels of environmental exposure of DTBBA.

Acute toxicity of DTBBA is low in mammals [2, 3], though longer term animal studies have not been conducted. The median lethal dose of DTBBA in rats following oral is greater than 4 g/kg, and is 10 g/kg in rabbits following dermal application [2, 3]. DTBBA is a potential skin sensitizer in guinea pigs, implying that DTBBA is reactive in skin although the extent of dermal absorption was not determined [4].

Due to the lack of adequate toxicity data, DTBBA was nominated to the National Toxicology Program for toxicity testing in rodents to help inform the potential human health impacts following environmental exposures. Metabolism and disposition data are essential for the design of toxicology studies and interpretation of toxicity data. However, there are no metabolism and disposition data for DTBBA in rodents. Therefore, a series of studies were undertaken to investigate the disposition and metabolism of DTBBA following oral administration and dermal application in male and female Harlan Sprague Dawley rats and B6C3F1/N mice (Table 1). Limited studies were also undertaken following intravenous (IV) administration of DTBBA in rats and mice to evaluate the absorption following oral and dermal routes. Additionally, *in vitro* work was conducted to aid mechanistic understanding of *in vivo* metabolism.

**Table 1:** Study design of [^14^C]DTBBA in Sprague Dawley rats and B6C3F1/N mice.

Species (Sex)	Dose (mg/kg)	Route	Study Duration (h)	Endpoint
Rat (M)	4, 40, 400	Oral	24	Dose response
Rat (M)	400	Oral	72	Dose response
Rat (F)	40	Oral	24	Sex difference
Rat (M)	4	Intravenous	24	Route difference
Rat (M)	4	Intravenous	24	Biliary excretion
Rat (M)	0.4, 4	Dermal^a^	72	Route difference
Mouse (M)	4, 40, 400	Oral	24	Species difference
Mouse (F)	40	Oral	24	Sex difference
Mouse (M)	4	Intravenous	24	Route difference
Mouse (M)	0.4, 4	Dermal^b^	72	Route difference

a Dermal site was covered.

## 2 Materials and methods

### 2.1 Chemicals and reagents

**Fig. 1:**
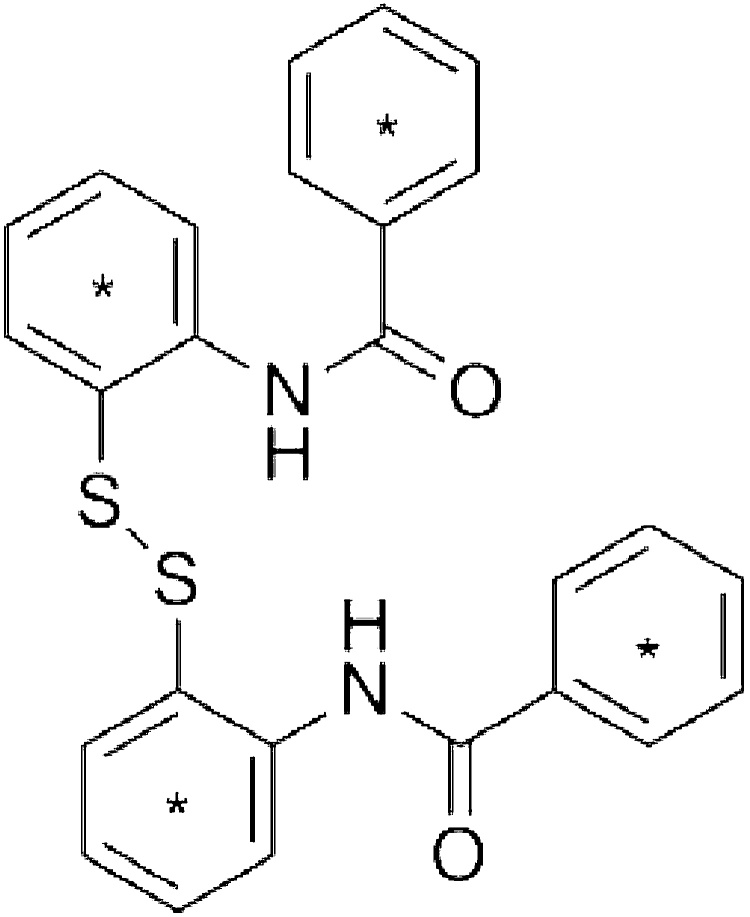
Structure of 2′,2′′′-dithiobisbenzanilide. * Denotes the radiolabeled rings.

2′,2′′′-dithiobisbenzanilide (CAS # 135-57-9; purity 95.4%) was obtained from Swan Chemical, Inc. (Lyndhurst, NJ). Uniformely ring labeled [^14^C]DTBBA (37 mCi/mmol) (Fig. 1) was obtained from Moravek Biochemicals, Inc. (Brea, CA). [^14^C]DTBBA was supplied as a solution in ethanol (0.1 mCi/mL). The radiochemical purity was 98% as determined by high performance liquid chromatography (HPLC) Method 1 (see below). Benzanalide, benzoate, and hippurate and were purchased from Sigma-Aldrich, Inc. (St. Louis, MO). 2-Phenylbenzothiazole standard was purchased from Alfa Aesar (Ward Hill, MA). Alkamuls EL-620 was purchased from Rhodia (Cranbury, NJ). Carbo-sorb E, Permafluor E^+^, and Ultima Gold scintillation cocktail were purchased from PerkinElmer (Boston, MA). In-Flow ES scintillation cocktail was obtained from IN\US Systems (Tampa, FL). Hydrogen peroxide (30%) was purchased from Fisher Scientific (Pittsburg, PA). All other reagents were purchased from commercial sources.

**HPLC Method 1**. An Agilent (Agilent Technologies, Santa Clara, CA) 1100 HPLC with a UV detector and an IN\US Systems β-RAM Model 3 radioactivity detector equipped with a lithium solid glass cell (500 μL) or a liquid cell (500 μL) was used with UV detection at 254 nm. Luna C18 column (150 x 4.6 mm, 5 μm) was used with mobile phase water:acetonitrile (45:55) run at 0.2 mL/min.

**HPLC Method 2.** Same instrumentation and detection systems was used as in HPLC Method 1 with a Varian Inertsil C8 column (250 x 4.6 mm, 5 μm) was used with mobile phases A (0.1% aqueous trifluoroacetic acid, TFA) and B (acetonitrile) run at 1.5 mL/min with a linear gradient from 0% B to 100% B over 20 min, and held for 5 min.

**HPLC Method 3**. Same instrumentation and detection systems was used as in HPLC Method 1 above with a Luna C18 column (4.6 x 150 mm, 5 μm) (Phenomenex, Inc., Torrance, CA). Mobile phases A (water) and B (acetonitrile) were run at 1 mL/min with a linear gradient from 10% B to 95% B over 15 min and held at 95% B for 5 min.

**HPLC Method 4.** Same system and detection systems as HPLC Method 3 was used with a Luna C18 column (250 x 10.0 mm, 5 μm) and a flow rate of 2 mL/min. Post-separation the column flow was split with the majority directed toward a collection vial, and a small portion was directed to the β-RAM for peak identification.

**MS Method 1**. A PE Sciex API 365 Triple Quad Mass Spectrometer (Foster City, CA) (Santa Clara, CA) was used with samples injected via direct infusion (50 μL/min) in positive ion electrospray ionization. The ion source temperature and ion spray voltage was 450 °C and 5500 V, respectively.

**MS Method 2.** A PE Sciex API 365 Triple Quad Mass Spectrometer coupled to an Agilent 1100 HPLC (Santa Clara, CA) or an AB Sciex API 5000 Triple Quad Mass Spectrometer (Applied Biosystems; Foster City, CA) coupled to an Agilent 1200 HPLC was operated in ESI positive ion mode (Applied Biosystems; Foster City, CA). A combination of full scan (*m/z* 50–2000), neutral loss scan or multiple reaction monitoring (MRM) was used to identify metabolites in urine. The MRM transitions monitored included the addition of oxygen, glucoronidation, sulfation, demethylation, removal of benzoic acid (Table S1). A Luna C18 column (150 x 4.6 mm, 5 μm Phenomenex, Inc., Torrance, CA) was used with mobile phases A (0.1% TFA) and B (acetonitrile/0.1% TFA) at 1 mL/min. The initial conditions were 20% B for 5 min then increased linearly to 100% B over 25 min and held at 100% B for 20 min. The mass spectrometer parameters were as follows: source temperature, 425 °C; ionspray voltage, 5500 V.

**MS Methood 3.** Accurate mass measurement of fractions was conducted by direct infusion using a Waters Micromass LCT Premier time-of-flight instrument (Milford, MA) in positive and negative ESI modes. The cone voltage was 75 V (positive and negative mode) and capillary voltage was 2800 V in positive mode or 2900 V in negative mode.

### 2.2 Nuclear magnertic resonace (NMR) spectroscopy

Proton NMR spectra of DTBBA and select isolated fractions were obtained using either a Bruker Avance 500 NMR Spectrometer (Billerica, MA) using CDCl_3_ as the solvent or a Bruker Avance-III 300 MHz NMR Spectrometer (Billerica, MA) using acetone-d6 as the solvent. The single dimension (1D) experiments consisted of ^1^H and ^13^C NMR. The two dimensional (2D) NMR experiments included proton-proton coupling via homonuclear correlation spectroscopy (COSY), single-bond proton-carbon coupling via heteronuclear single-quantum correlation spectroscopy (HMQC), and 3-bond proton-carbon coupling via heteronuclear multiple-bond correlation spectroscopy (HMBC). Experiments were conducted using standard program parameters available with instrument software. Estimates of proton and carbon chemical shifts were made with MarvinSketch 6.0.2 (Marvin 6.0.2, ChemAxon (http://www.chemaxon.com).

### 2.3 Animals

All animal studies were approved by the Lovelace Respiratory Research Institute’s Institutional Animal Care and Use Committee, conducted in facilities accredited by the Association for Assessment and Accreditation of Laboratory Animal Care International, and carried out in compliance with the Guide for the Care and Use of Laboratory Animals (NRC, 1996). Adult male (207–283 g) and female (147–171 g) Sprague Dawley rats, either intact or surgically pre-fitted with bile duct cannulae, were purchased from Harlan Laboratories, Inc (Livermore, CA and Indianapolis, IN). Adult male (22–31 g) and female (19–22 g) B6C3F1/N mice were obtained from Taconic Laboratories (Germantown, NY). Animals were 8–13 weeks old at the time of dosing. The animals were quarantined for at least 6 days before they were used in a study, and were randomized into dosing groups and group housed (n = 3) in shoebox cages with hardwood bedding. The room temperature was maintained at 18–28 °C and relative humidity was maintained within 30–70%. A 12-h light:dark cycle was maintained during the quarantine and study periods. During studies, animals were housed individually in all-glass Roth style metabolism chambers that provided for separate collection of urine, feces, CO_2_, and expired volatile organic chemicals (VOCs). Animals were fed certified, irradiated NTP 2000 feed (Zeigler Brothers, Inc.; Gardeners, PA) and Kirtland Air Force base municipal water was available *ad libitum*. Feed was used within 180 days of the milling date.

### 2.4 Adminsitration of DTBBA in sprague Dawley rats and B6C3F1/N mice

Male or female rats or mice were given a single oral (target dose of 4, 40, or 400 mg/kg), dermal (target dose of 0.4 or 4 mg/kg), or IV (target dose of 4 mg/kg) dose. All dose formulations contained [^14^C]DTBBA and unlabeled DTBBA to achieve the final desired DTBBA concentration and specific activity. The target radioactivity per animal was (100 μCi/kg in mouse and 50 μCi/kg in rat). Oral dose formulations were prepared as suspensions in aqueous 0.5% methylcellulose and administered by intragastric oral at 5 mL/kg to rats and 10 mL/kg to mice. The IV formulations were prepared in dimethylacetamide:polyethylene glycol 400:saline (15:55:30, v:v:v) using [^14^C]DTBBA and administerd via a lateral tail vein at a dose volume of 1 mL/kg to rats and 4.5 mL/kg to mice. Dermal dose formulations were prepared in acetone and applied at 0.5–1 mL/kg to rats and mice. Approximately 18 h prior to dermal dosing, hair from the intrascapular region was clipped from an area no less than 2 cm x 2 cm. If any cuts or abrasions were observed, the animals were replaced. The dose site (1 cm x 1 cm) was marked with felt tip marker and the dose area protected from grooming by attachment of a metal mesh stainless steel isolator (Shandon Lipshaw Micro Cassette #331 positive clip, LabX Scientific Marketplace) using methylacrylate cement (Super Glue Corporation/Pacer Technology; Rancho Cucamunga, CA). Doses were applied with a 100 μL Luer lock gas-tight syringe through the mesh to the dose the marked dose site, the solvent allowed to evaporate, and animal was returned to the metabolism cage.

The concentration of [^14^C]DTBBA in the dose formulations was determined by liquid scintillation spectroscopy (LSS) analysis of weighed aliquots of formulations collected before, during, and after dosing for a total of five aliquots sampled on the day of dosing. Radiochemical purity of each formulation was assessed using HPLC Method 1 on the day of dosing and was ≥ 96%.

### 2.5 Collection of biological samples and analysis for radioactivity

All samples were stored at −20 °C until analysis. Urine collections were made on ice at 4, 8, 12, 24 h and at 24-h intervals, as appropriate, following dose administration.The urine from the bladder at necropsy was added to the final urine collection. At the end of each collection interval, the metabolism cages were rinsed with water (or ethanol after the terminal collection only) and collections were stored separately. Feces was collected at 24 h-intervals following dose administration. Expired VOCs and CO_2_ were collected at intervals similar to urine by passing air through the metabolism chambers and then through traps (first 2 traps contained isopropanol to collect VOCs and the second 2 placed in line contained 1 N NaOH to collect CO_2_). All traps were cooled on wet ice. Bile was collected from bile duct cannulated rats over the intervals 0–1, 1–2, 2–4, 4–8, 8–12, and 12–24 h after dose administration

At the end of all in life experiments (24 or 72 after dose adminditration), animals were anesthesized with sodium pentobarbital/phenytoin, and blood collected via cardiac puncture using heparinized syringes and then euthanized by section of the diaphragm. Prior to necropsy in dermal studies, the dermal isolator (and glue attached) was removed from the back of the animal and wiped with guazesoaked in chlorohexidine soap. Toluene was used to remove all methacrylate glue was removed from the dermal isolator and skin surrounding the dose site. The dermal dose site skin was then wiped three times with chlorohexidine and all wipes collected for analysis. The area was then dried with a clean gauze and the dermal site skin was excised and stored. At necropsy, the following tissues were collected: adipose (perirenal, reproductive), muscle (hind leg, trapezius), and skin (ears), as well as the following in their entirety: adrenals, brain, lung, heart, spleen, pancreas, kidneys, testes or uterus and ovaries, liver, thyroid, thymus, stomach, small intestine, cecum, large intestine (all gastrointestinal (GI) tract tissues without contents), and urinary bladder. Contents from the stomach, small intestine, large intestine and cecum were combined and weighed. GI tract tissues were rinsed with deionized water following removal of contents and the rinsate was combined with GI tract contents.

Triplicate aliquots of urine, cage rinse, CO_2_, VOCs, and bile were mixed with Ultima Gold scintillation cocktail for determination of radioactivity. Feces were homogenized with an approximately equal amount of water. Triplicate aliquots of feces homogenates and tissue aliquots (∼ 50–150 mg) (except GI tract tissues, skin and carcass) were transferred to weighed oxidizer cones for oxidation using a Packard 307 Biological Sample Oxidizer (Shelton, CT) using Carbo-sorb E for trapping of CO_2_ and Permafluor E^+^ as the scintillation cocktail. Larger whole tissues (i.e., liver, brain, kidney) were homogenized and then aliquots were weighed into oxidizer cones prior to oxidation. Samples of GI tissue, whole carcass, GI contents, skin (including dermal dose site) were weighed, digested in 2 N ethanolic sodium hydroxide and aliquots were transferred into Ultima Gold scintillation cocktail for determination of radioactivity. All samples were analyzed for total radioactivity using a PerkinElmer Model 2500 TR (Shelton, CT) LSS. Total weights for the dispersed tissues were calculated using the body weight for rats as adipose 7.0%, blood 7.4%, muscle 40.4%, and skin 19% and mice as adipose 7.0%, blood 4.9%, muscle 38.4%, and skin 16.5% [5].

### 2.6 DTBBA-derived radioactivity associated with liver *in vivo*

The method of Masson et al. [6] was used to determine radioactivity associated with macromolecules in the liver of rats and mice 24 h following oral administration of [^14^C]DTBBA (400 mg/kg). Aliquots of liver tissue (∼50 mg) were homogenized using a mortar and pestle homogenizer in a 1.5 mL Eppendorf tube with 1 mL of a 1:1 mixture of methanol:water and the mixture was centrifuged at 12,000 rpm for 5 min. The supernatant was collected and the remaining pellet was homogenized with 1 mL of 3:1 dichloromethane:methanol, centrifuged as above and the supernatant was collected. This two-step extraction procedure was repeated until no more radioactivity was detected in the supernatant. After the extraction, the pellet was oxidized and analyzed for radioactivity by LSS. Any radioactivity detected in the tissue pellet was presumed to be associated with tissue macromolecules.

### 2.7 Reaction of DTBBA with glutathione *in vitro*

The potential for DTBBA to react with protein thiols were investigated *in vitro*. DTBBA (5.35 mM in ethanol, 100 μL) and GSH (26.75 mM in water, 100 μL) were stirred for 30 min at room temperature and analyzed by HPLC Method 2. The peaks were collected, the solvent was removed under vacuum, and the residue was reconstituted in 1 ml of methanol:10 mM aqueous ammonium acetate (1:1, v:v). Samples were analyzed by MS Method 1.

### 2.8 Metabolite profiling and identification

Composite urine samples were prepared for each dose group and filtered with a 0.45 μm filter and profiled using HPLC Method 3. Following initial profiling, fractions were isolated using HPLC Method 4. Post-separation the column flow was split with the majority directed toward a collection vial, and a small portion was directed to the β-ram for peak identification. Samples were dried and resuspended in methanol:10 mM ammonium acetate (1:1). Pooled urine samples and fractions were analyzed by MS Methods 2 and 3. Fractions were also analyzed by NMR spectroscopy.

## 3 Results

Full data sets for all disposition groups can be found at https://manticore.niehs.nih.gov/cebssearch/paper/14786/private/ADME22DITH.

### 3.1 Disposition of [^14^C]DTBBA following oral and intravenous administration and dermal application in rats

A group of male rats (400 mg/kg) carried through to 72 h following a single oral dose administration showed that the excretion was fast and complete by 24 h (Table 2) and hence all subsequent oral dose groups were taken to only 24 h following dose adminsitration. Following a single oral administration of [^14^C]DTBBA to male rats at 4, 40, and 400 mg/kg, the majority of the administered dose was excreted in urine (∼ 44−66%) and feces (21−29%) 24 h following administration (Table 2 and Table S2). Excretion as CO_2_ or VOCs was negligible. The pattern of excretion was similar following a 40 mg/kg dose in male and female rats (Table 2). Following IV administration to male rats at 4 mg/kg, the pattern of excretion was similar to oral except that there was less dose excreted in feces following IV administration (16%) compared to an equivalent oral dose (∼29%) (Table 2). Following IV administration of [^14^C]DTBBA (4 mg/kg) to rats fitted with indwelling biliary cannulae, ∼ 13% of the dose was recovered in bile 24 h post dose; in addition ∼ 7% excreted in feces suggesting direct excretion of radioactivity into the intestinal lumen via gut epithelium (Table 2). These data suggest that at a significant portion of the dose excreted in feces following oral administration is likely due to a combination of biliary excretion and direct excretion into the intestinal lumen via gut epithelium rather than poor absorption.

**Table 2:** Disposition of radioactivity 24 h following oral and intravenous (IV) administration of [^14^C]DTBBA in rats.

	Percent dose recovered^a^						
Sample	4 mg/kg (Male, Oral)	40 mg/kg (Male, Oral)	400 mg/kg (Male, Oral)	400 mg/kg (Male, Oral, 72 h)	40 mg/kg (Female, Oral)	4 mg/kg Male (Male, IV)	4 mg/kg Male (Male, IV)
Urine^b^	44.2 ± 7.81	51.3 ± 6.70	66.1 ± 28.2	70.2 ± 3.1	57.6 ± 1.50	73.1 ± 7.00	ND
Feces	29.0 ± 10.8	24.3 ± 8.40	21.1 ± 7.80	36.8 ± 23.3	28.1 ± 17.8	16.1 ± 5.8	7.19 ± 1.36
GI Content	4.61 ± 2.32	2.34 ± 0.93	1.93 ± 1.32	0.0524 ± 0.0261	3.07 ± 1.17	1.13 ± 0.55	
Bile	ND	ND	ND	ND	ND	ND	13.1 ± 3.5
Volatile Organics	0.0326 ± 0.0075	ND^c^	0.0968 ± 0.0095	0.168 ± 0.021	0.0843 ± 0.0082	0.0630 ± 0.0413	ND
CO_2_	0.320 ± 0.094	ND	0.747 ± 0.053	1.40 ± 0.16	0.633 ± 0.061	0.447 ± 0.275	ND
Carcass	0.0592 ± 0.1323	0.151 ± 0.127	0.322 ± 0.202	0.138 ± 0.077	0.237 ± 0.155	0.0767 ± 0.108	ND
Tissues	0.631 ± 0.292	0.763 ± 0.102	0.910 ± 0.427	0.366 ± 0.078	0.717 ± 0.238	3.56 ± 2.68	ND
Total Recovered^d^	79.1 ± 16.4	78.8 ± 5.7	91.0 ± 32.9	109 ± 23.0	90.5 ± 18.8	93.5 ± 5.80	ND

a Mean ± SD for N = 4 animals.

b Includes cage rinse and bladder contents at necropsy.

c ND = Not determined.

d Excludes the percent dose recovered in carcass.

The recovery of radioactivity in tissues was low and similar across doses and sexes 24 h following oral (< 1%) and IV administrations (< 4%) (Table 2). In male rats following oral administration of 400 mg/kg and scarificed at 72 h (0.910% at 24 h and 0.366% at 72 h, Table 2), the total radioactivity remained in tissues decreased by about 50% suggesting tendency for low tissue retention of administered dose. Tissue distribution of radioactivity is shown in Table 3. The bladder and GI tract tissues tended to have a higher concentration including some apparent sex difference (i.e.higher bladder concentration in males compared to females) which is likely due to contamination from excreta. When the 40 mg/kg male and groups are compared, although adrenal and thymus concentrations in males were ∼ 3-fold higher than corresponding concentrations in females, it is difficult to determine the biological relevance due to either the high vaiability or the low concentrations found. The thyroid concentration in males (6.94 ± 3.72) were ∼ 38-fold higher than that in females (0.181 ± 0.404); although this highlights some biological significance, the reason for the observed difference in unclaer at the present time. The highest tissue/blood ratio (TBR) was observed in liver and kidney. Thyroid TBR was also significant in male rats at doses up to 40 mg/kg but were lower at the highest dose of 400 mg/kg. A high TBR in thyroid was not observed in the female rats receiving oral dose of [^14^C]DTBBA.

**Table 3:** Tissue distribution of radioactivity 24 h following oral and intravenous (IV) administration of [^14^C]DTBBA in rats.

Concentration (nmol-eq DTBBA per g tissue)^a^					
Tissue	4 mg/kg (Male, Oral)	40 mg/kg (Male, Oral)	400 mg/kg (Male, Oral)	40 mg/kg (Female, Oral)	4 mg/kg (Male, IV)
Blood	0.0446 ± 0.0157	0.485 ± 0.209	5.93 ± 1.87	0.318 ± 0.120	0.0710 ± 0.0152
Adipose	0.0513 ± 0.0405	0.653 ± 0.403	5.39 ± 3.19	0.259 ± 0.174	0.0414 ± 0.0153
Muscle	0.0156 ± 0.0185	0.190 ± 0.0470	1.59 ± 0.49	0.0535 ± 0.0478	0.0257 ± 0.0134
Skin	0.0430 ± 0.0211	0.620 ± 0.2110	6.60 ± 3.83	0.862 ± 0.0435	0.108 ± 0.052
Brain	0.00217 ± 0.00285	0.0714 ± 0.0189	1.13 ± 0.30	0.0146 ± 0.0134	0.0142 ± 0.0062
Heart	0.0150 ± 0.0119	0.247 ± 0.0620	2.22 ± 0.94	0.116 ± 0.029	0.0314 ± 0.0050
Kidneys	0.198 ± 0.0880	2.18 ± 1.00	15.9 ± 7.8	1.42 ± 0.100	0.259 ± 0.066
Liver	0.172 ± 0.0500	1.56 ± 0.390	15.0 ± 7.1	1.06 ± 0.015	0.243 ± 0.059
Lung	0.0402 ± 0.0156	0.530 ± 0.155	3.83 ± 1.29	0.379 ± 0.057	20.4 ± 29.7
Spleen	0.0181 ± 0.0152	0.326 ± 0.084	2.65 ± 1.17	0.128 ± 0.033	0.527 ± 0.167
Adrenals	0.0631 ± 0.0432	1.61 ± 1.66	9.37 ± 2.06	0.471 ± 0.318	0.109 ± 0.054
Thymus	0.00751 ± 0.00874	0.125 ± 0.051	1.78 ± 0.54	0.0451 ± 0.0321	0.0170 ± 0.0072
Thyroid	0.332 ± 0.552	6.94 ± 3.72	11.1 ± 7.9	0.181 ± 0.404	0.322 ± 0.140
Urinary Bladder	0.706 ± 0.504	9.11 ± 10.6	23.8 ± 13.9	0.615 ± 0.578	0.519 ± 0.394
Pancreas	0.0459 ± 0.0410	0.560 ± 0.283	6.15 ± 4.63	0.288 ± 0.079	0.160 ± 0.250
Testes	0.00978 ± 0.01234	0.121 ± 0.025	1.46 ± 0.56	0.392 ± 0.131	0.0216 ± 0.0130
Cecum	1.01 ± 0.550	10.2 ± 2.60	60.9 ± 39.3	4.96 ± 2.92	0.579 ± 0.297
Large Intestine	0.469 ± 0.214	5.82 ± 0.920	26.6 ± 15.5	3.6 ± 2.14	0.258 ± 0.214
Small Intestine	0.513 ± 0.282	4.87 ± 1.17	55.3 ± 40.3	3.83 ± 0.930	0.336 ± 0.132
Stomach	0.534 ± 0.225	4.22 ± 1.79	32.8 ± 29.0	3.88 ± 5.02	0.126 ± 0.084

a Mean ± SD for N = 4 animals.

Following dermal application to male rats, ∼ 40 and 16% of 0.4 mg/kg and 4.0 mg/kg, respectively, was absorbed with ∼ 16% and 3.5% of the applied dose remaining at the dose site skin, 72 h following application (Table 4). The main routes of excretion were via urine and feces, continuing to the 72 h terminal time point. Total residual radioactivity in tissues was ∼ 6% and ∼ 5% of the administered dose, respectively for 0.4 and 4 mg/kg (Table 4). Tissue distribution of radioactivity is given in Table 5. At 4 mg/kg, the concentration in skin, muscle and thyroid was higher than in other tissues except in bladder and GI tract tissues where the concentration observed was high likely due to contamination from excreta.

**Table 4:** Disposition of radioactivity 72 h following dermal application of [^14^C]DTBBA in male rats.

	% Dose recovered^a^	
	0.4 mg/kg	4 mg/kg
Total Unabsorbed Dose^b^	30.9 ± 23.9	53.4 ± 19.9
Dose Site Skin	16.2 ± 13.0	3.49 ± 1.49
Absorbed Dose^c^	39.5 ± 17.7	16.3 ± 12.7
Urine^d^	8.99 ± 3.45	3.94 ± 4.47
Feces	21.2 ± 9.2	6.40 ± 6.45
GI Content	2.75 ± 1.61	0.99 ± 1.53
Tissues	6.21 ± 4.81	4.58 ± 1.92
Carcass	ND^f^	ND
Total Recovered^e^	86.6 ± 21.8	73.2 ± 23.7

a Mean ± SD for N = 4 animals.

b Sum of the dose recoiverd in dose site rinses and appliance.

c Sum of excreta and tissues.

d Includes cage rinse and bladder contents at necropsy.

e Excludes the percent dose recovered in carcass.

f ND, not detected.

**Table 5:** Tissue distribution of radioactivity 72 h following dermal application of [^14^C]DTBBA to male rats and mice.

Concentration (nmol-eq DTBBA per g Tissue)^a^				
	Rat		Mouse	
	0.4 mg/kg	4 mg/kg	0.4 mg/kg	4 mg/kg
Blood	0.00797 ± 0.00424	0.0407 ± 0.0117	0.0171 ± 0.0096	0.0138 ± 0.0036
Adipose	0.00927 ± 0.00245	0.0346 ± 0.0117	0.0113 ± 0.0045	0.0243 ± 0.0048
Muscle	0.00433 ± 0.00101	0.356 ± 0.480	0.0135 ± 0.0071	0.0206 ± 0.0072
Skin	0.238 ± 0.211	1.22 ± 0.36	0.0312 ± 0.0207	0.436 ± 0.619
Brain	0.00283 ± 0.00027	0.0290 ± 0.0201	0.00551 ± 0.00306	0.00483 ± 0.00146
Heart	0.00895 ± 0.00070	0.0157 ± 0.0057	0.00761 ± 0.00321	0.0115 ± 0.0034
Kidneys	0.00777 ± 0.00188	0.0507 ± 0.0383	0.00753 ± 0.00174	0.0376 ± 0.0195
Liver	0.00696 ± 0.00219	0.0469 ± 0.0232	0.0153 ± 0.0137	0.0353 ± 0.0169
Lung	0.00956 ± 0.00060	0.0398 ± 0.0448	0.00589 ± 0.00220	0.0125 ± 0.0027
Spleen	0.00309 ± 0.00044	0.0192 ± 0.0062	0.00819 ± 0.00456	0.0118 ± 0.0058
Adrenals	0.0237 ± 0.00691	0.0737 ± 0.0273	0.0697 ± 0.0129	0.291 ± 0.293
Thymus	0.00438 ± 0.00053	0.0296 ± 0.0287	0.0171 ± 0.0074	0.0190 ± 0.0106
Thyroid	0.00720 ± 0.00229	0.202 ± 0.153	0.0604 ± 0.0435	0.0842 ± 0.0177
Urinary Bladder	0.0612 ± 0.0290	0.260 ± 0.375	0.149 ± 0.142	0.131 ± 0.100
Pancreas	0.00368 ± 0.00067	0.0316 ± 0.0125	0.00855 ± 0.00088	0.0265 ± 0.0113
Testes	0.00737 ± 0.00075	0.0147 ± 0.0029	0.00956 ± 0.00546	0.00925 ± 0.00129
Cecum	0.109 ± 0.106	0.551 ± 0.607	0.0406 ± 0.0317	0.134 ± 0.075
Large Intestine	0.0437 ± 0.0446	0.160 ± 0.192	0.0222 ± 0.0107	0.0630 ± 0.0186
Small Intestine	0.0158 ± 0.00321	0.199 ± 0.202	0.0165 ± 0.0079	0.0777 ± 0.0369
Stomach	0.0191 ± 0.010	0.0879 ± 0.0671	0.0242 ± 0.0289	0.0455 ± 0.0200

a Mean ± SD for N = 4 animals.

### 3.2 Disposition of [^14^C]DTBBA following oral and intravenous administration and dermal application in mice

Following a single oral administration of [^14^C]DTBBA to male mice at 4, 40, or 400 mg/kg, the majority of radioactivity was excreted in urine (∼29−61%) and feces (∼16−45%) 24 h after administration (Table 6). Excretion as CO_2_ was ∼ 1.5–5% and as VOCs was ∼0.5-2.0%. The amount of radioactivity recovered in urine increased with dose up to 400 mg/kg. Although there was corresponding decerase in feces, a trend was not evident likely due to contamination of feces with urine in the mid dose group. The pattern of excretion was similar following equivalent doses in male and female mice (Table 6). Following a single IV administration of [^14^C]DTBBA to male mice at 4 mg/kg, the pattern of excretion was similar to oral (Table 6).

**Table 6:** Disposition of radioactivity 24 h following oral and intravenous (IV) administration of [^14^C]DTBBA in mice.

	Percent dose recovered^a^				
Matrix	4 mg/kg (Male, Oral)	40 mg/kg (Male, Oral)	400 mg/kg (Male, Oral)	40 mg/kg (Female, Oral)	4 mg/kg (Male, IV)
Urine^b^	28.7 ± 12.4	30.7 ± 9.8	60.9 ± 14.5	32.6 ± 5.2	42.7 ± 11.4
Feces	27.9 ± 3.7	44.7 ± 20.1	15.9 ± 6.0	28.9 ± 9.8	38.0 ± 10.9
GI Contents	0.26 ± 0.15	7.92 ± 0.29	0.246 ± 0.165	0.192 ± 0.071	0.255 ± 0.086
Volatile Organics	0.479 ± 0.072	2.04 ± 2.77	0.823 ± 0.335	0.709 ± 0.072	0.188 ± 0.028
CO_2_	3.92 ± 0.58	1.53 ± 0.28	4.97 ± 0.81	4.53 ± 0.23	2.05 ± 1.08
Carcass	0.0562 ± 0.0336	5.22 ± 1.168	0.100 ± 0.076	0.394 ± 0.509	1.45 ± 2.98
Tissues	0.575 ± 0.198	0.810 ± 1.219	0.588 ± 0.136	0.911 ± 0.473	3.58 ± 3.92
Total Recovered^c^	62.5 ± 15.4	81.0 ± 21.4	85.3 ± 15.7	72.4 ± 9.0	88.1 ± 10.5

a Mean ± SD for N = 4 animals.

b Includes cage rinse and bladder contents at necropsy.

c Excludes the percent dose recovered in carcass.

The residual radioactivity in tissues at 24 h following oral and IV administration was <1% and 3.6%, respectively (Table 6). Tissue distribution of radioactivity is shown in Table 7. The tissues of excretion tended to have a higher concentration of residual radioactivity possibly due to contamination from excreta. The highest TBR was observed in liver, thyroid, and thymus.

**Table 7:** Tissue distribution of radioactivity 24 h following oral and intravenous (IV) administration of [^14^C]DTBBA in mice.

Concentration (nmol-eq DTBBA per g tissue)^a^					
Tissue	4 mg/kg (Male, Oral)	40 mg/kg (Male, Oral)	400 mg/kg (Male, Oral)	40 mg/kg (Female, Oral)	4 mg/kg (Male, IV)
Blood	0.0400 ± 0.0088	0.298 ± 0.126	2.38 ± 0.64	0.429 ± 0.130	0.166 ± 0.102
Adipose	0.0271 ± 0.0080	20.7 ± 45.9	2.27 ± 0.63	0.262 ± 0.048	0.0329 ± 0.0063
Muscle	0.0162 ± 0.0033	0.130 ± 0.032	2.38 ± 1.06	0.161 ± 0.035	0.0211 ± 0.0022
Skin	0.0371 ± 0.0090	0.221 ± 0.092	3.85 ± 1.08	0.382 ± 0.143	0.0431 ± 0.0105
Brain	0.0157 ± 0.0098	0.105 ± 0.051	0.931 ± 0.282	0.138 ± 0.018	0.0118 ± 0.0052
Heart	0.0249 ± 0.0025	0.148 ± 0.041	1.59 ± 0.37	0.261 ± 0.030	0.0562 ± 0.0260
Kidneys	0.0488 ± 0.0093	0.367 ± 0.051	4.80 ± 1.05	0.434 ± 0.107	0.151 ± 0.037
Liver	0.120 ± 0.006	0.777 ± 0.123	12.3 ± 0.8	1.53 ± 0.35	0.290 ± 0.020
Lung	0.0319 ± 0.0053	0.172 ± 0.086	2.69 ± 0.64	0.440 ± 0.201	0.116 ± 0.078
Spleen	0.0180 ± 0.0040	0.167 ± 0.093	1.69 ± 0.78	0.211 ± 0.095	0.985 ± 1.034
Adrenals	0.397 ± 0.190	1.73 ± 1.39	56.2 ± 57.2	3.24 ± 0.78	0.151 ± 0.060
Thymus	0.103 ± 0.172	0.227 ± 0.187	2.74 ± 0.87	0.267 ± 0.114	0.0317 ± 0.0131
Thyroid	0.147 ± 0.071	0.600 ± 0.466	12.5 ± 7.5	1.43 ± 0.55	0.310 ± 0.198
Urinary Bladder	0.140 ± 0.097	0.918 ± 1.224	12.4 ± 9.2	1.22 ± 0.42	0.130 ± 0.045
Pancreas	0.0211 ± 0.0054	0.177 ± 0.043	23.9 ± 48.9	0.139 ± 0.034	0.0305 ± 0.0082
Testes	0.0207 ± 0.0059	0.113 ± 0.059	2.37 ± 1.56	0.360 ± 0.078	0.0159 ± 0.0027
Cecum	0.148 ± 0.084	0.750 ± 0.217	7.1 ± 29.8	0.861 ± 0.201	0.322 ± 0.076
Large Intestine	0.0557 ± 0.0058	0.360 ± 0.077	36.3 ± 59.9	0.407 ± 0.089	0.181 ± 0.050
Small Intestine	0.0465 ± 0.0099	0.347 ± 0.052	16.7 ± 10.7	0.305 ± 0.133	0.140 ± 0.045
Stomach	0.102 ± 0.053	0.506 ± 0.137	15.1 ± 9.9	0.614 ± 0.190	0.0527 ± 0.0162

a Mean ± SD for N = 4 animals.

Following dermal application in ∼50 and ∼ 11% of 0.4 mg/kg and 4.0 mg/kg, respectively, was absorbed with ∼ 2–3% of the dose recovered at the dose site skin, 72 h (Table 8). Main routes of excretion were via urine and feces. Mice also excreted significant amount radioactivity as CO_2_ (∼14% of dose) following application of 0.4 mg/kg. The higher dose group (4 mg/kg) was performed first without collection of VOCs and CO_2_ because they were not expected to be present. Due to low recovery of radioactivity in the 4 mg/kg, the VOC and CO_2_ collections were added at the 0.4 mg/kg dose. Total residual radioactivity in tissues was ∼1-2% for both dose groups (Table 8). The tissue distribution data for both dose groups is shown in Table 5. As with other route, the tissues of excretion tended to have a higher concentration of residual radioactivity possibly due to contamination from excreta.

**Table 8:** Disposition of radioactivity 72 h following dermal application of [^14^C]DTBBA in male mice.

	% Dose recovered^a^	
	0.4 mg/kg	4 mg/kg
Total Unabsorbed Dose^b^	33.9 ± 2.7	72.3 ± 3.8
Dose Site Skin	2.09 ± 0.51	2.55 ± 1.35
Absorbed Dose^c^	49.8 ± 8.7	10.6 ± 2.9
Urine^d^	12.4 ± 4.3	3.73 ± 1.49
Feces	14.4 ± 3.2	5.39 ± 2.28
CO_2_	13.5 ± 11	ND^e^
GI Content	0.324 ± 0.122	0.225 ± 0.076
Carcass	0.00 ± 0.00	0.018 ± 0.040
Tissues	1.55 ± 0.68	0.981 ± 1.131
Total Recovered^f^	85.9 ± 9.1	85.4 ± 5.0

a Mean ± SD for N = 4 animals.

b Sum of the dose recoiverd in dose site rinses and appliance.

c Sum of excreta and tissues.

d Includes cage rinse and bladder contents at necropsy.

e ND, not determined.

f Excludes the percent dose recovered in carcass.

### 3.3 DTBBA-derived radioactivity associated with liver and *in vitro* reaction of DTBBA with glutathione

At 24 h, following a 400 mg/kg oral dose, 32.1 ± 8.8% and 37.9 ± 12.9% of radioactivity in liver was covalently associated with liver macromolecules in rat and in mouse, respectively. In order to investigate the ineraction of DTBBA with thiol groups of protein, DTBBA was incubated with GSH. Three peaks were detected in these incubations at 9.7 (A), 14.9 (B), and 18.5 (C) min (vs DTBBA at 18.8 min). Mass spectrum of peak A contained a dominant ion at *m/z* 535 and upon MS/MS fragmentation had product ions observed at *m/z* 460 and 406 (Figure S1A). These data suggested that Peak A was a disulfide product between glutathione and *N*‐(2‐sulfanylphenyl)benzamide, which is the disulfide cleavage product of DTBBA. Peak B eluted with a retention time of 14.9 min which corresponds to an ion at *m/z* 212 (Figure S1B). This ion matches the retention time of authentic *N*‐(2‐sulfanylphenyl)benzamide and likely an ion corresponding to loss of water from *N*‐(2‐sulfanylphenyl)benzamide. When Peak B (*N*‐(2‐sulfanylphenyl)benzamide) was isolated and allowed to stand at 37 °C it converted to Peak C. Peak C had a retention time of 18.5 min and showed a predominant ion at *m/z* 212 and MS/MS product ion at *m/z* 109 which corresponds to the loss of the C_6_H_5_CN moiety (Figures S1C and D). An authentic standard of 2-phenylbenzothiazole had a retention time of 18.5 min and had identical MS spectrum as Peak C (data not shown). Based on these data the products were assigned the structures as shown in Figure S-1A-D.

### 3.4 Metabolite profiling and identification

**Fig. 2:**
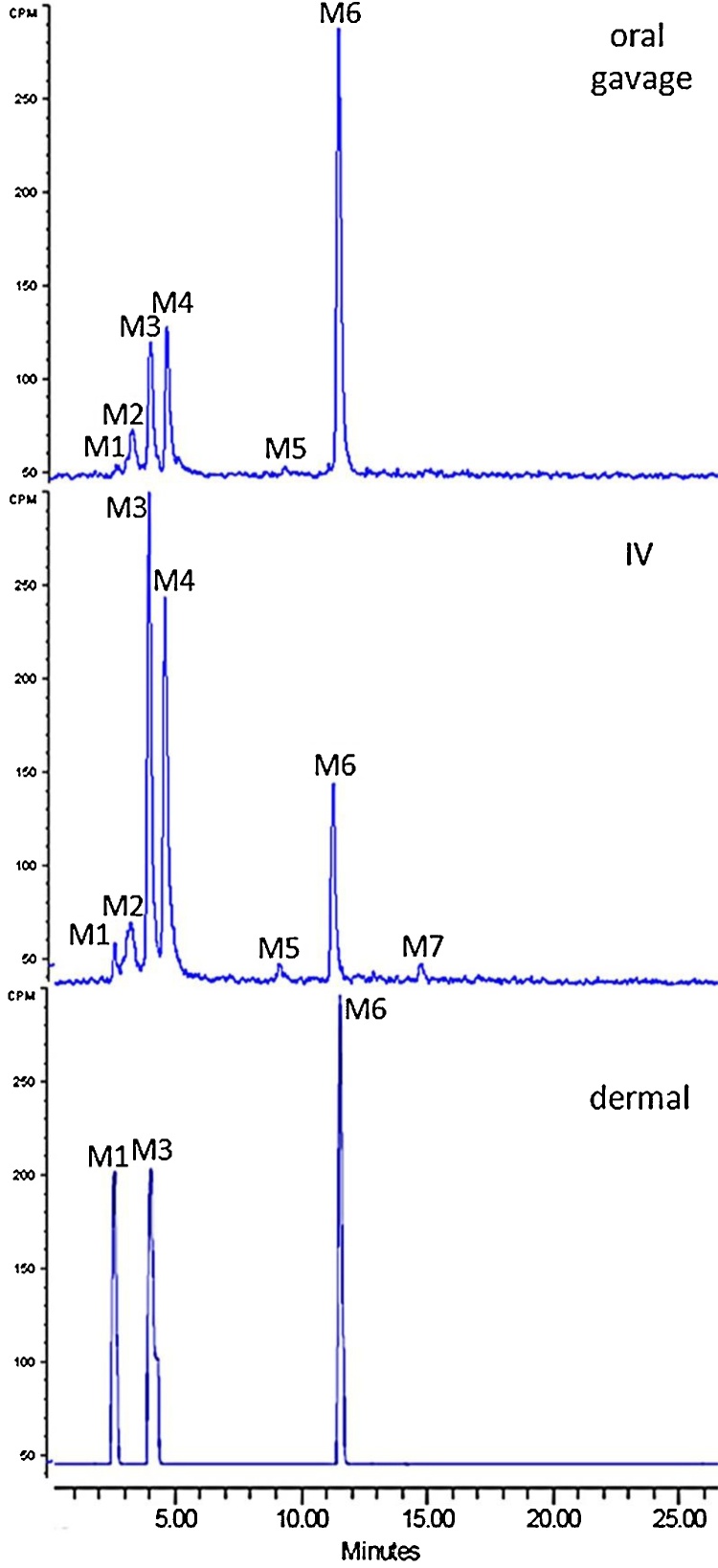
HPLC radiochromatogram of rat urine collected 0–4 h following administration of 4 mg/kg [^14^C]DTBBA to male rats by various routes.

The profiles of rat or mouse urine following multiple routes of adminsitration consisted of four major (M1, M3, M4, and M6) and three minor peaks (M2, M5, and M7) with percent in chromatogram varying with route, species or the dose. Representative chromatograms for male rat urine are shown in Fig. 2 and the distribution of radioactivity in individual HPLC radiochromatograms are summarized in Table S2. The predominant peak after oral administration and dermal application in rats was M6, which accounted for 33–74% of total radioactivity in chromatogram. The metabolites M3 (∼36% of chromatogram) and M4 (∼ 34% of chromatogram) were predominant in urine after IV administration. Mouse urine metabolic profiles were similar to those observed in rat urine, and M6 was also the predominant urinary metabolite in this species (Table S2). There was no apparent sex differences in the urine profile in rats or mice (data not shown). Overall, there did not appear to be major differences in urinary profiles following oral administration or dermal application in both species.

The LC/MS/MS analysis using MRM allowed the assignment of potential structures for M1–M5, which are shown in Figure S2. Based on MRM transitions, metabolites M1, M2, M4, and M5 were indicated as oxygenated metabolites (+16). The transition for hydroxylated DTBBA (473.2 → 121.0 *m/z*) at 7.3 min was observed in MRM data but not present above background in the radiochromatogram. Cleavage of one or more anilide bond was indicated for M1, M3, and M5. Transitions associated with DTBBA were detected at 25.5 min though below levels detectable by radiochromatography. HPLC-UV coelution experiments were performed with pure standards of benzanalide, benzoate or hippurate with radiochromatogram peaks. A small peak (M7) coeluted with benzanalide at 15.3 min (Data not shown).

**Fig. 3:**
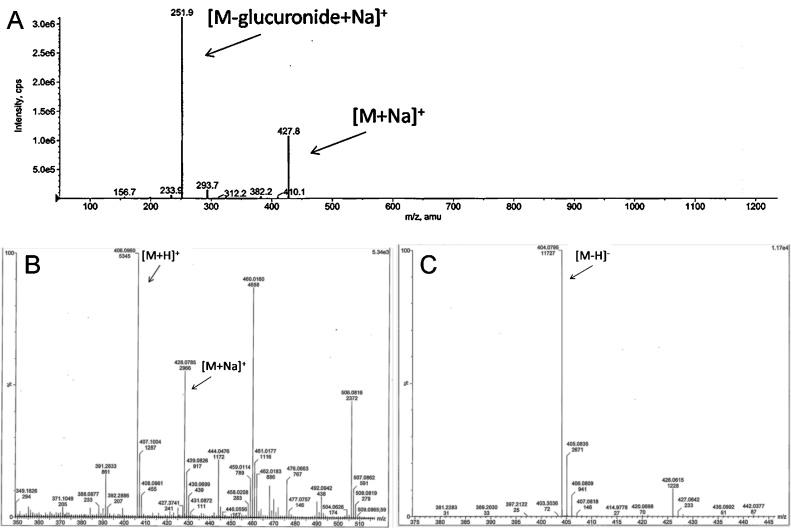
Analysis of urine metabolite M6 by mass spectrometty in positive ion ESI-MS A) MS spectrum of metabolite M6 showing sodium adduct (*m/z* 428) and an ion corresponding to the loss of glucuronidemoiety (*m/z* 251) (B) positive ion MS/MS spectrum of metabolite M6 (C) negative ion MS/MS spectrum of metabolite M6.

**Fig. 4:**
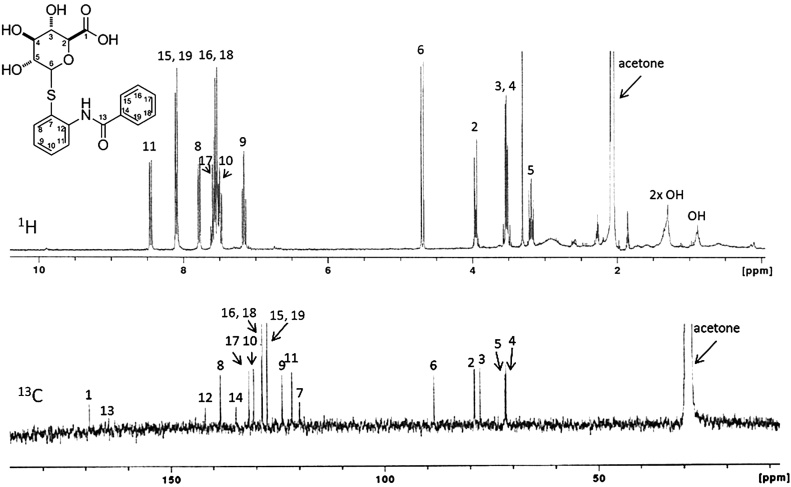
^1^H and ^13^C NMR spectra and signal assignments of urinary metabolite M6.

Urine collected 0−8 h after a 400 mg/kg oral dose to male rats was pooled and used for isolation and collection of the predominant urine metabolite M6. Initial LC–MS analysis via positive ionization showed a [M+H]^+^ ion with *m/z* 406.1 and in negative mode [M−H]^−^ ion with *m/z* 404.1 (Fig. 3B and C). ESI-MS/MS spectrum of *m/z* 428 ([M + Na]^+^) gave a loss of 176, suggesting a glucuronide conjugate (Fig. 3A). Accurate mass spectrum of M6 yielded a *m/z* of 406.0960 which corresponds well with the calculated value of *m/z* 406.0960 for the glucuronide with the formula C_19_H_20_NO_7_S. Taken together, these data suggest that M6 to be a glucuronide of the disulfide bond cleavage product of DTBBA, *N*-(2-sulfanylphenyl)benzamide. To determine the position of glucuronidation both ^1^H and ^13^C NMR spectra were obtained (Fig. 4). Additional 2 dimensional spectral data including proton-proton coupling (COSY), single-bond proton-carbon coupling (HMQC), and 3-bond proton-carbon coupling (HMBC) and assignments are shown in Table S3. Overall measured spectral data met predicted chemical shifts in both 1H and 13C. Peak multiplicity from ^1^H spectra and ^1^H-^13^C correlations from 2D experiments supported assignment of structure to *N*-(2-sulfanylphenyl)benzamide. The expected chemical shifts of the DTBBA-derived metabolite were not expected to vary significantly between an *N*- or *S*-glucuronide. However the chemical shifts of the glucuronide moiety and the thiol were major indicators of position of attachment of the glucuronide. Critical to assignment of glucuronide position of attachment was the presence or absence of the chemical shift of the thiol proton. An *N*-substituted glucuronide would leave a free thiol with a proton estimated chemical shift of ∼3.7 ppm. No resonance at this position was detected.The expected chemical shift of the proton on the glucuronic acid at the thioether carbon ∼4.5 ppm correlated well with that measured (4.7 ppm). Long range proton correlation measured via HMBC between carbon 6 (glucuronide thioether carbon) and carbon 7 (benzene carbon adjacent to thiol) confirmed the glucuronidation at the thiol. Metabolite M6 was determined to be an *S*-linked glucuronide *N*-(2-glucuronylsulfanylphenyl)benzamide; the conjugated disulfide link cleavage product of DTBBA.

## 4 Discussion

The literature is devoid of information regarding metabolism and disposition of DTBBA, a molecule that has significant use in rubber production and therefore may pose exposure risk to workers in this field. To the best of our knowledge, this is the first report investigating the metabolism and disposition of DTBBA in rodents.

Based on the dose recovered in urine and tissues, the oral absorption of DTBBA in rodents was estimated to be at least 60% of the dose administered. However, the observed excretion in feces and bile following IV administration and in feces following dermal adminiatrtaion suggest that some of the administered dose excreted in feecs following oral adminditration is likely from the absorbed dose. Taking this into consideration, ∼ 60% may represent a minimum value and that absorption may be as high as ∼ 80% following oral adminstration. DTBBA was also moderately absorbed (∼ 40–50%) following dermal application of 0.4 mg/kg [^14^C]DTBBA in rats and mice: the % dose absorbed decreased with the increasing dose with a value of ∼ 11–16%. There was no apparent species or sex difference in absorption of DTBBA following oral administration or dermal application.

Regardless of dose and route of administration, the main routes of excretion of DTBBA were via urine and feces in both rats and mice. Excretion following oral administration was rapid and the majority of the dose was excreted within 24 h in both rats and mice. In rats, the excretion of radioactivity in urine seems to increase with the dose with a concomitant decrease in the feces although due to higher variability in data, likely due to contamination of feces with urine, it is difficult to confirm this pattern. Following dermal application, the majority of the absorbed dose was excreted in urine and feces within 72 h. Excretion as CO_2_ was negligible in rats, but this represents an important pathway in mice following both oral and dermal routes. Mice excreted up to 5% and 13% of the applied dose as CO_2,_ respectively, following oral administration and dermal application of [^14^C]DTBBA. This suggests a potential species difference in metabolism as a ring opening is necessary to release CO_2_ although with the data available at the present time,w e are unable to confirm this.

Biliary excretion of DTBBA equivalents was significant. In male rats following IV administration of 40 mg/kg [^14^C]DTBBA, 13% of the dose was excreted in bile at 24 h with approximately 7% recovered in feces. These data suggest that direct excretion of DTBBA equivalents into the intestinal lumen via gut epithelium may contribute to nearly a third of fecal clearance of systemic DTBBA equivalents, while biliary excretion accounts for the remaining two-thirds. Typically, secretion via intestinal tissues is driven by transporter-mediated transepithelial flux of the xenobiotic [7].

The percent dose retained in tissue at 24 h following oral administration was <1% in both rats and mice indicating low potential for tissue accumulation. Following dermal application in rats up to 6% of the applied dose was recovered in tissues although in mice it was much lower (up to 1.5%). The highest TBRs were observed in liver and kidney of male and female rats; thyroid was also showing a high TBR at doses up to 40 mg/kg, but were ∼2 at the highest dose of 400 mg/kg. The high TBR in thyroid was not observed in the female rats receiving oral doses of [^14^C]DTBBA. In male mice, liver had the highest TBR.

Profiling of rat and mouse urine showed that DTBBA was highly metabolized: no parent DTBBA was detected. Radiochromatograms consisted of seven postulated metabolites; four major and three minor. The predominant metabolite, which accounted for more than 50% of the radiochemical content in urine, was confirmed as glucuronide of the disulfide link cleavage product, *N*-(2-sulfanylphenyl)benzamide, using a combination of mass spectrometry and NMR spectroscropy. Overall, there did not appear to be major differences in the metabolic profile between rats and mice following single doses of DTBBA delivered via oral or dermal routes. Dose did not appear to have significant impact on the urine metabolic profile either quantitatively or qualitatively in either species.

The *in vivo* ubiquity of sulfur and thiol containing molecules, such as oxidized and reduced glutathione (GS-SG, GSH, respectively), and cysteine would suggest that the disposition and metabolism of DTBBA may be influenced by the chemistry of these sulfides. *N*-(2-sulfanylphenyl)benzamide was detected when DTBBA was incubated in vitro with GSH and following administration of DTBBA in rodents likely via the formation of *N*-(2-sulfanylphenyl)benzamide/glutathione adduct. This adduct may then be available for further metabolism or exchange with another molecule of GSH, yielding GS-SG and *N*-(2-sulfanylphenyl)benzamide. This reaction has been demonstrated with molecules with similar disulfide structure. For example Ellman’s reagent, 5,5′-dithiobis-(2-nitrobenzoic acid) (DTNB), reacts stoichiometrically with thiols and is used to quantify the number or concentration of thiols in tissues and other biological matrices. Thiols of proteins and other biomolecules reduce DTNB, cleaving the disulfide bond to yield equimolar amounts of the thiol, 3-thio-6-nitrobenzoate [8,9]. Administration of the cancer chemotherapeutic agent dimesna (2,2′-disulfanediyldiethanesulfonic acid) to rats results in the release of the monomer mesna and formation of a GSH/mesna dimer [10,11]. This GSH exchange reaction is likely to be in liver or kidney (Goren et al. [11]. With DTBBA, this is demonstrated by the presence of DTBBA equivalents associated with rat liver suggesting covalent interactions of DTBBA or its metabolites with thiols or other nucleophilic sites in proteins. The disulfide bond of DTBBA may also be reduced in plasma since *N*-(2-sulfanylphenyl)benzamide was detected within 1 h of incubation of DTBBA with rat plasma during pilot studies (data not shown).

The principal driver of the reactivity of a thiol via a thiol/disulfide exchange is the pKa [12]. The pKa of the thiol of N-(2-sulfanylphenyl)benzamide freed by interaction of DTBBA with glutathione (and potentially other sulfhydryl groups in vivo), is ∼6 vs cysteine or glutathione (∼10) and thus the predominant form at pH 7.4 would be the thiolate (>85%). The free thiol form of the cardiovascular drug JTT-705 forms mixed disulfide adducts to protein and GSH. Mesna (2-mercaptoethanesulfonate) forms dimers with glutathione via this mechanism [11]. We hypothesize that DTBBA equivalents bound to protein in liver and other tissues may behave in an analogous manner to that described above for JTT-705 and dimesna. Initially, thiols from tissue proteins or GSH reacts with DTBBA releasing the thiolate containing metabolite *N*-(2-sulfanylphenyl)benzamide. The *N*-(2-sulfanylphenyl)benzamide may then in turn reduce tissue and cytosolic disulfides via thiol/disulfide reaction. Another possibility would be by disulfide linkage fission via radical formation [13] and subsequenty reacting with thiol radicals.

Other metabolites of DTBBA in urine were products of hydrolysis, oxidation, and Phase II metabolism based on LC–MS/MS analysis. Small amounts of *N*-(2-sulfanylphenyl)benzamide (M7) and the major urinary metabolite (M6), identified as the S-glucuronide of *N*-(2-sulfanylphenyl)benzamide suggests that the cleavage of DTBBA occurs *in vivo*. This unusual S-glucuronide was detected as the predominant metabolite in rat urine and bile. The pharmaceutical agent JTT-705, which possesses an 2-aminobenzene-1-thiol core similar to DTBBA, is metabolized by *S*-glucuronide formation in a manner similar to the formation of *N*-(2-sulfanylphenyl)benzamide from DTBBA [14].

**Fig. 5:**
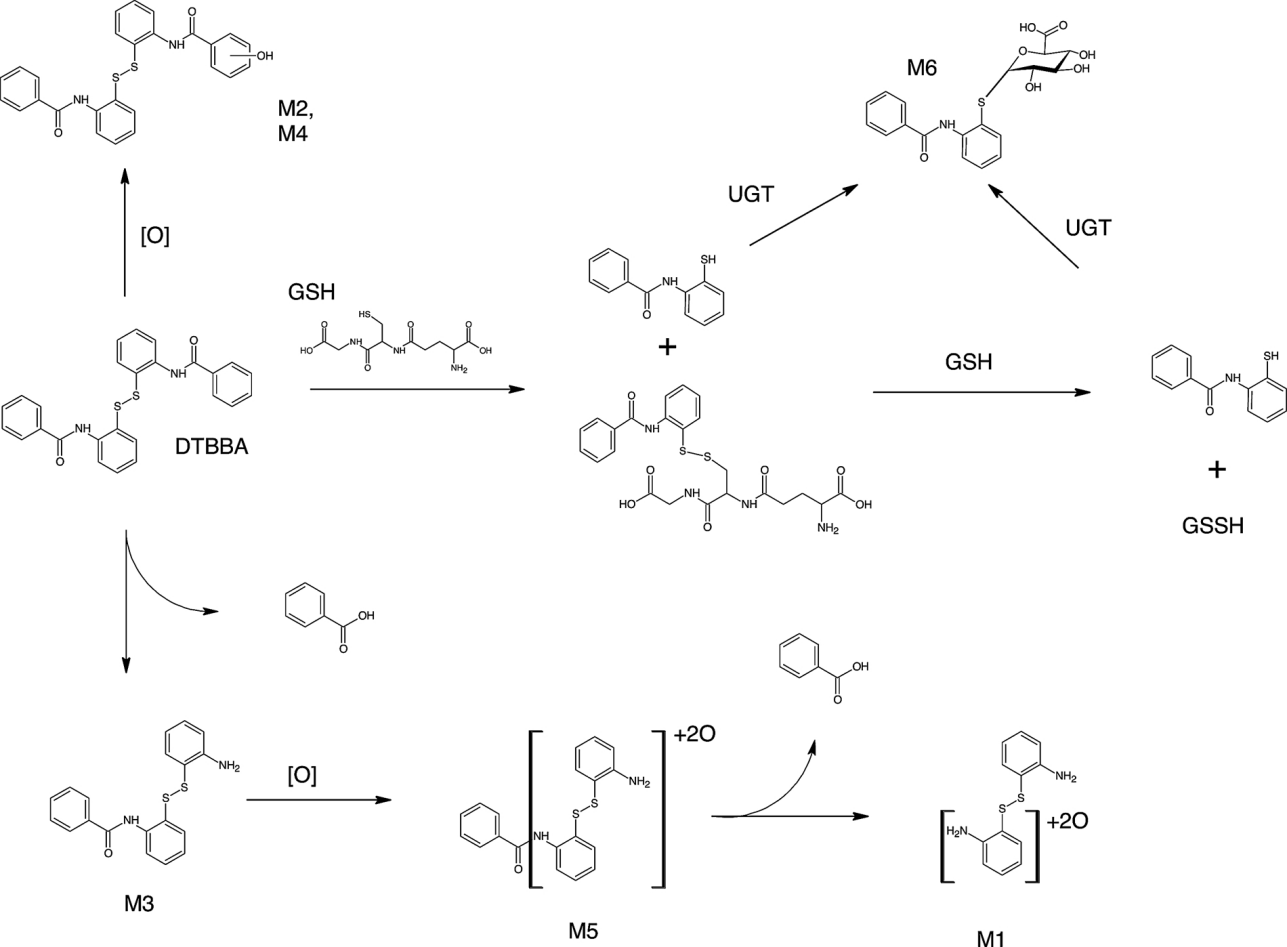
Proposed metabolism of DTBBA in rodents.

Additional metabolites were tentatively identified using MRM transitions. A hydroxylation product M2 was identified. The benzoic acids of DTBBA appear to be sequentially removed either through hydrolysis or via oxidative metabolism, and then the core structure is sequentially oxidized (M2, M3, and M4). Any released benzoate would be conjugated with glycine to yield hippuric acid, which is quantitatively eliminated in rat urine [15]. However, we were unable to detect radiolabeled benzoate or hippurate in urine samples collected in these studies. The production of CO_2_ derived from aromatic ring label by mice is of great interest as this represents a pathway of metabolism not observed in rat. Regardless of the route of administration of [^14^C]DTBBA, mice generated CO_2_, converting as much as 13% of applied dose when 4 mg/kg was applied dermally. One possibility may be conversion of benzoate to CO_2._ Unlike rat, mice only eliminate *ca.* 50% of administered benzoic acid as hippurate, presumably eliminating the remainder via oxidation [15]. Fig. 5 shows the proposed metabolism of DTBBA in rodents based on these evaluations and identification of urinary metabolites following administration of DTBBA.

## 5 Conclusion

These studies suggest that DTBBA is absorbed both orally and dermally in rodents and that its ADME properties are generally conserved across rodent species, sex, and dose level. Via its principal pathway of metabolism, DTBBA interacts with GSH or other thiol containing moieties, suggesting formation of GSH disulfide conjugates and freeing the thiobenzanilide moiety, which may interact further with free cysteines in the cytosol or are conjugated with glucuronic acid.

Acknowledgements

The authors are grateful to Mr. Bradley Collins and Dr. Esra Mutlu for review of this manuscript. This work was supported by the Intramural Research Program of the NIH, National Institute of Environmental Health Sciences, Intramural Research project ZIA ES103316-04, and performed for the National Toxicology Program, National Institute of Environmental Health Sciences, National Institutes of Health, U.S. Department of Health and Human Services, under contract HHSN291200775562C (Lovelace Respiratory Research Institute, Albuquerque, New Mexico).

## Declaration of Competing Interest

The authors report no conflicts of interest.
